# Influenza vaccine coverage and factors associated with non-vaccination among adults at high risk for severe outcomes: An analysis of the Canadian Longitudinal Study on Aging

**DOI:** 10.1371/journal.pone.0275135

**Published:** 2022-09-30

**Authors:** Katie Gravagna, Christina Wolfson, Giorgia Sulis, Sarah A. Buchan, Shelly McNeil, Melissa K. Andrew, Jacqueline McMillan, Susan Kirkland, Nicole E. Basta

**Affiliations:** 1 Department of Epidemiology, Biostatistics and Occupational Health, School of Population and Global Health, McGill University, Montreal, QC, Canada; 2 Neuroepidemiology Research Unit, Research Institute of the McGill University Health Centre, Montreal General Hospital, Montreal, QC, Canada; 3 Health Protection, Public Health Ontario, Toronto, ON, Canada; 4 Dalla Lana School of Public Health, University of Toronto, Toronto, ON, Canada; 5 Division of Infectious Diseases, Department of Medicine, Dalhousie University, Halifax, NS, Canada; 6 Division of Geriatrics, Department of Medicine, Dalhousie University, Halifax, NS, Canada; 7 Division of Geriatric Medicine, Departments of Medicine and Community Health Sciences, University of Calgary, Calgary, AB, Canada; 8 Division of Geriatric Medicine, Department of Community Health and Epidemiology, Dalhousie University, Halifax, NS, Canada; Taipei Medical University, TAIWAN

## Abstract

**Background:**

Influenza vaccination is recommended in Canada for older adults and those with underlying health conditions due to their increased risk of severe outcomes. Further research is needed to identify who within these groups is not receiving influenza vaccine to identify opportunities to increase coverage.

**Objectives:**

We aimed to 1) estimate influenza non-vaccination prevalence and 2) assess factors associated with non-vaccination among Canadian adults aged ≥65 and adults aged 46–64 with ≥1 chronic medical condition (CMC) due to their high risk of severe influenza outcomes.

**Methods:**

We conducted a secondary analysis of cross-sectional data collected from 2015–2018 among participants of the Canadian Longitudinal Study on Aging. For both groups of interest, we estimated non-vaccination prevalence and used logistic regression models to identify factors associated with non-vaccination. We report adjusted odds ratios and 95% confidence intervals for the investigated variables.

**Results:**

Overall, 29.5% (95% CI: 28.9%, 30.1%) of the 23,226 participants aged ≥65 years and 50.4% (95% CI: 49.4%, 51.3%) of the 11,250 participants aged 46–64 years with ≥1 CMC reported not receiving an influenza vaccination in the past 12 months. For both groups, lack of recent contact with a family doctor and current smoking were independently associated with non-vaccination.

**Discussion:**

Influenza vaccination helps prevent severe influenza outcomes. Yet, half of adults aged 46–64 years with ≥1 CMC and more than one-quarter of all adults aged ≥65 years did not receive a recommended influenza vaccine in the year prior to the survey. Innovation in vaccination campaigns for routinely recommended vaccines, especially among those without annual family doctor visits, may improve coverage.

**Conclusion:**

Influenza vaccination coverage among Canadian adults aged 46–64 years with ≥1 CMC and adults aged ≥65 years remains suboptimal. Vaccination campaigns targeting those at high risk of severe outcomes without routine physician engagement should be evaluated to improve uptake.

## 1. Introduction

Although influenza is typically perceived as a mild illness, there are an estimated 5.7 million hospitalizations for influenza-associated lower respiratory infections and approximately 250,000–500,000 deaths worldwide each year, and some estimates suggest that global influenza-associated mortality exceeds half a million deaths each year [1, 2]. Canada alone has an estimated 3,500 deaths and 12,200 hospitalizations annually, and the combined category of influenza and pneumonia remains in the top ten causes of death nationwide [3, 4].

Annual influenza vaccination of groups at high risk of severe outcomes [5] is recommended as part of immunization services in Canada [6] and in many other countries where influenza vaccines are widely available [7]. For all Canadian provinces except Quebec, influenza vaccination recommendations are presented at the national level by the National Advisory Committee on Immunization (NACI) [8], and influenza vaccination is recommended and funded for anyone aged 6 months or older without contraindications as of the 2021–2022 season [6, 9]. In Quebec, the *Comité sur l’immunisation du Québec* provides the influenza vaccination recommendations for the province [8], and influenza vaccination is only funded for certain groups at high risk of severe influenza outcomes or influenza transmission, such as those aged 60 years and older and healthcare workers [6, 9]. The National Advisory Committee on Immunization (NACI) "*particularly* recommends" influenza vaccination for adults with at least one chronic medical condition (CMC) and older adults (those aged 65 years and older) due to their high risk for influenza-related complications [3].

Despite the provision of publicly funded influenza vaccines [9] and the significant benefits compared to low risk [10, 11] of vaccination [3, 4], influenza vaccination coverage rates across Canada have remained suboptimal for these groups at high risk of severe outcomes. In 2005, limited progress on increasing influenza vaccination rates led to a National Consensus Conference on Vaccine-Preventable Diseases in Canada recommendation to develop a vaccination coverage target of 80% by 2025 [3, 12]. Vaccination among people 65 years and older was estimated to be 65% during the 2015/2016 season, 69% during the 2016/2017 season, and 70.7% during the 2017/2018 season [3, 13, 14]. Low influenza vaccination coverage was especially pronounced for those aged under 65 with at least one CMC; this group had vaccination coverage estimated as low as 37% for the 2015/2016 and 2016/2017 influenza seasons, and only 39.4% during the 2017/2018 season [3, 13, 14]. More recently, during the 2019/2020 season, coverage in Canada slightly increased to 44% in those aged under 65 with at least one CMC, while coverage in those aged 65 and older was 70% [15].

The Canadian Longitudinal Study on Aging (CLSA) presents an ideal platform for investigating multiple aspects of influenza vaccination given the broad and comprehensive data collected on a wide range of factors including influenza vaccine uptake along with a self-reported diagnosis of CMC (type and number), sociodemographic factors, healthcare utilization, and self-rated health and health behaviors [16].

Identifying those who are recommended to but who are not receiving influenza vaccine within these high-risk groups could help public health programs develop new vaccination campaign approaches. Vaccination has the potential to prevent hospitalizations and deaths; therefore, this knowledge will help us to better understand and monitor influenza vaccine uptake and ideally intervene effectively to increase uptake among those at high risk of severe outcomes who are not receiving influenza vaccine.

In this study, we aimed to 1) estimate the prevalence of influenza non-vaccination between 2015–2018 and 2) assess factors associated with non-vaccination among Canadian adults. We assessed these aims among two groups that would benefit from influenza vaccination: adults aged 65 years and older and adults aged 46–64 years with at least one chronic medical condition.

## 2. Materials and methods

### 2.1 Data source

The CLSA is a national study of 51,338 adults aged 45–85 at enrollment (2011–2015), recruited from all 10 Canadian provinces. The CLSA includes two cohorts that differ in mode and scope of data collection: the Comprehensive cohort (N = 30,097 at baseline), which provides a core set of data plus more in-depth data collected in person, and the Tracking cohort (N = 21,241 at baseline), which provides a core set of data collected by telephone only. Participants were recruited using 3 sampling frames: the Canadian Community Health Survey–Healthy Aging (CCHS), random-digit dialing, and provincial health registries. Participants were randomly selected from within 25–50 km of 11 data collection sites in 7 provinces in the Comprehensive cohort and were randomly selected across all 10 provinces in the Tracking cohort [17].

Participants were not eligible to participate in the CLSA if they were a resident of a federal First Nations reserve or other First Nations settlement; a full-time member of the Canadian Armed Forces; a resident in the 3 territories; living in long-term care institutions; unable to respond in French or English; or cognitively impaired. More information on the CLSA study procedures and participant recruitment can be found elsewhere [16–18]. Data collection occurs every 3 years among all participants; the follow up 1 (FUP1) visit data were collected from 2015–2018 [17]. Additionally, the CLSA baseline and FUP1 protocol and other supporting documentation are available on the CLSA website (clsa-elcv.ca).

CLSA participants provided consent for data collection and data analysis for research when enrolling in the CLSA. This analysis of fully de-identified secondary data from the CLSA did not require separate participant consent. Ethics review and approval for this analysis was obtained from the McGill University Institutional Review Board (Application Number: 21-02-048), and access to these data in accordance with all CLSA guidelines was obtained from the CLSA (Application Number: 2006029).

### 2.2 Analytic sample

We conducted a secondary analysis of cross-sectional data obtained from 44,815 CLSA participants during FUP1—Comprehensive (N = 27,765) and Tracking (N = 17,050). To be eligible for this analysis, participants had to have participated in FUP1 and reported whether or not they had received influenza vaccine in the past 12 months. Our analyses combined the data from both Comprehensive and Tracking participants and included only those variables that were collected from all participants.

### 2.3 Assessing influenza vaccination coverage and factors associated with influenza vaccine uptake

The variables included in these analyses were identified *a priori* as those of potential public health interest relevant to targeted vaccination campaigns and/or those previously cited in the literature as potentially associated with influenza vaccination in similar contexts as enumerated below [19–22]. In addition to CLSA FUP1 data, data about participants’ race and education level were obtained from the CLSA baseline assessment. The CLSA survey questions, variables, response options, and variable categorizations used in the analysis can be found in the appendix (**S1 Table**).

#### 2.3.1 Outcome

Our primary outcome of interest was self-reported influenza vaccination within the past 12 months, based on the survey question “Have you had… Flu shot in the last 12 months”. Respondents who answered “no” were categorized as not having been vaccinated in the past 12 months and are referred to as non-vaccinated throughout. Respondents who answered “yes” were categorized as having been vaccinated in the past 12 months and are referred to as vaccinated throughout. Participants who responded, “don’t know/no answer”, who “refused”, or had a “missing” response to this question were aggregated in the dataset and were excluded from the analysis. The use of self-reported influenza vaccination status has been validated as a method with high sensitivity and moderate specificity compared to medical records for older adults [23, 24].

#### 2.3.2 Sociodemographic variables

To assess individual factors potentially associated with non-vaccination in those at high risk of severe outcomes, the independent variables of age, sex at birth, race, education, household income, province of residence, and rural or urban classification were evaluated.

#### 2.3.3 Chronic medical conditions

To evaluate vaccination uptake among those with CMCs, respondents who self-reported a physician diagnosis of any of the following conditions were considered to have at least one CMC based on NACI’s definition of groups for whom influenza vaccination is particularly recommended: heart disease, heart attack or myocardial infarction, high blood pressure or hypertension, respiratory disorders, kidney disease or failure, asthma, diabetes, cancer, dementia or Alzheimer’s disease, Parkinsonism or Parkinson’s Disease, stroke or cerebrovascular accident (CVA), or ministroke or Transient Ischemic Attack (TIA) [25]. Heart disease, respiratory disorders, kidney disease or failure, asthma, diabetes, and cancer were coded as separate binary variables. All other CMCs listed were combined into the variable “other CMC”. The new variable “number of CMC” was derived by summing and categorizing the number of “yes” responses to each of the binary CMC and “other CMC” variables for each participant.

#### 2.3.4 Healthcare utilization, health perception, and health behaviors

Healthcare utilization factors—hospitalization history, contact with a family doctor, and specialist contact within the past 12 months—were also evaluated for potential association with influenza vaccination. Participant responses were dichotomized for each variable (**S1 Table**).

Since health status and the limitations and challenges associated with aging have been consistently identified as factors associated with influenza vaccination in Canada and the United States, including for those at high risk of severe outcomes [26–28], we included self-rated health in our analyses. Self-rated health has been validated [29, 30] as a measure of health in general health surveys.

The presence of limitations that lead to the need for assistance with daily activities may impede the ability to receive influenza vaccination, particularly in older adults. Therefore, we identified respondents who reported receiving professional or non-professional care in the past 12 months. Participants who received both professional and non-professional care were included in both variables for analysis.

Participants were also asked about their current smoking behavior, alcohol consumption in the past 12 months, and exercise in the past 7 days (**S1 Table**), as these health behaviors were found to be associated with influenza vaccination in a 2004 publication of the older (≥65 years) adult Canadian population using data from the Canadian Study of Health and Aging [26].

### 2.4 Statistical analysis

We estimated the proportion of self-reported influenza non-vaccination in the previous 12 months for adults aged 65 years and older and adults aged 46–64 years with at least one CMC overall and within each group stratified by the variables within the categories of sociodemographics, CMC, healthcare utilization, health perception, and health behaviors.

We also used multivariable logistic regression models to estimate the association between the independent variables and influenza non-vaccination for those aged 65 years and older and aged 46–64 years with at least one CMC. We reported adjusted odds ratios (aOR) for the association between each covariate included in each model and not having received influenza vaccine in the past 12 months, along with 95% confidence intervals (95% CIs).

In multivariable logistic regression model 1, we first estimated the associations between influenza non-vaccination and sociodemographic factors (such as age group and sex) and type of CMC (e.g., heart disease) due to their consistent association with influenza vaccination in previous studies. In model 2, we estimated the associations between influenza non-vaccination and additional measures of health status (number of CMC (0, 1, or ≥2)), received professional care (such as professional medical or personal care), received non-professional care (such as non-professional assistance with activities), contact with family doctor, contact with medical specialist, and hospitalization history in addition to the sociodemographic and type of CMC variables included in model 1. Finally, in model 3, our full model, we estimated the associations between influenza non-vaccination and self-reported and/or indirect measures of health (self-rated health, moderate or strenuous exercise frequency, smoking frequency, alcohol use) and the number residing in household in addition to the covariates in model 2.

Participants with missing values for one or more variables used in this analysis were excluded in the analyses involving those variables. The variable with the highest percentage of missing values among all participants with a valid response to the dependent variable was 6.6% for household income (**S1 Table**). Responses of “Don’t Know/No Answer”, “Refused”, “Missing”, and “Did not complete a DCS [Data Collection Site] visit” were categorized as missing.

The CLSA uses complex sampling techniques and provides inflation and analytic weights for the baseline datasets. However, sampling weights for FUP1 were not available from the CLSA at the time of the analyses, and CLSA baseline weights are not applicable for use with the FUP1 data. We did stratify by age and included the variables sex and province in our analyses as recommended by the CLSA [31]. More information on the use of weights is available from the CLSA [31].

Sensitivity analyses (using two-sample tests of proportions) were performed to see if the time frame in which respondents were asked about influenza vaccination was associated with being more likely to report being vaccinated. We compared the prevalence of vaccination among participants who were surveyed during any of the typical Canadian influenza seasons in 2015–2018 (e.g., Nov. 1 to April 30 in each year) to those who were surveyed outside of any typical annual influenza season across all survey years (May 1 to Oct 31 in each year) by cohort [3, 32].

The analyses were conducted in R version 1.3.1073.

## 3. Results

### 3.1 Participant demographics

The Comprehensive cohort included 27,765 participants who participated in FUP1, of whom 0.31% (N = 86) were excluded due to missing data for the outcome variable; the Tracking cohort included 17,050 participants who participated in FUP1, of whom 2.09% (N = 357) were similarly excluded. **Tables 1 and 2** present the demographic characteristics of those aged 65 years and older and those aged 46–64 years with at least one CMC by vaccination status. Among the 44,815 participants who participated in FUP1, 23,226 (51.8%) were aged 65 years and older at the time of FUP1 and 11,250 (25.1%) were aged 46–64 years with at least one CMC.

**Table 1:** Demographic characteristics by influenza vaccination status: Participants aged 65 years and older (N = 23,226).

	Received Influenza Vaccination in Last 12 Months (N = 16,373)		Did Not Receive Influenza Vaccination in Last 12 Months (N = 6,853)	
	N	%	N	%
Sociodemographics				
Age (years)				
65–74	8591	65.1	4606	34.9
75–84	6378	77.2	1888	22.8
85–94	1404	79.6	359	20.4
Missing	0	0.0	0	0.0
Sex				
Female	8127	69.8	3513	30.2
Male	8241	71.2	3331	28.8
Missing	5	0.03	9	0.1
Race				
White	15841	70.8	6541	29.2
Non-White	521	63.6	298	36.4
Missing	11	0.1	14	0.2
Education				
Less than secondary school graduation	1336	67.2	653	32.8
Secondary school graduation, no post-secondary education	1792	67.8	850	32.2
Some post-secondary education	1282	69.3	569	30.7
Post-secondary degree/diploma	11920	71.5	4751	28.5
Missing	43	0.3	30	0.4
Household Income (Canadian dollars)				
< 20000	764	58.7	537	41.3
≥20000-<50000	4690	66.5	2367	33.5
≥50000-<100000	6266	73.2	2293	26.8
≥100000-<150000	2190	76.5	674	23.5
≥150000	1080	74.5	370	25.5
Missing	1383	8.4	612	8.9
Province of Residence				
Newfoundland	962	65.8	500	34.2
Prince Edward Island	386	76.1	121	23.9
Nova Scotia	1709	81.5	387	18.5
New Brunswick	405	71.6	161	28.4
Quebec	2546	59.0	1770	41.0
Ontario	3910	75.7	1257	24.3
Manitoba	1451	71.0	592	29.0
Saskatchewan	375	68.9	169	31.1
Alberta	1650	73.6	593	26.4
British Columbia	2975	69.6	1298	30.4
Missing	1166	6.6	452	6.2
Urbanicity				
Urban	14160	71.3	5707	28.7
Rural	2199	65.9	1138	34.1
Missing	14	0.1	8	0.1

**Table 2:** Demographic characteristics by influenza vaccination status: Participants aged 46–64 years with at least one chronic medical condition (CMC) (N = 11,250).

	Received Influenza Vaccination in Last 12 Months (N = 5,583)		Did Not Receive Influenza Vaccination in Last 12 Months (N = 5,667)	
	N	%	N	%
Sociodemographics				
Age (years)				
46–54	1222	42.9	1624	57.1
55–64	4361	51.9	4043	48.1
Missing	0	0.0	0	0.0
Sex				
Female	2935	51.3	2784	48.7
Male	2645	47.9	2880	52.1
Missing	3	0.1	3	0.1
Race				
White	5261	49.8	5313	50.2
Non-White	316	47.5	349	52.5
Missing	6	0.1	5	0.1
Education				
Less than secondary school graduation	183	42.3	250	57.7
Secondary school graduation, no post-secondary education	544	46.8	618	53.2
Some post-secondary education	424	50.8	410	49.2
Post-secondary degree/diploma	4424	50.2	4383	49.8
Missing	8	0.1	6	0.1
Household Income (Canadian dollars)				
< 20000	259	48.5	275	51.5
≥20000-<50000	761	46.0	895	54.0
≥50000-<100000	1750	48.9	1829	51.1
≥100000-<150000	1215	48.9	1272	51.1
≥150000	1347	54.4	1130	45.6
Missing	251	4.5	266	4.7
Province of Residence				
Newfoundland	363	46.4	420	53.6
Prince Edward Island	105	53.6	91	46.4
Nova Scotia	579	65.1	311	34.9
New Brunswick	141	50.9	136	49.1
Quebec	729	33.1	1476	66.9
Ontario	1359	53.9	1160	46.1
Manitoba	489	51.1	468	48.9
Saskatchewan	126	50.0	126	50.0
Alberta	688	57.4	511	42.6
British Columbia	1002	50.9	967	49.1
Missing	372	6.2	353	5.9
Urbanicity				
Urban	4837	50.6	4720	49.4
Rural	743	44.0	946	56.0
Missing	3	0.1	1	0.02

### 3.2 Influenza non-vaccination in adults aged 65 years and older

The proportion of participants aged 65 years and older (N = 23,226) who reported not receiving influenza vaccination in the past 12 months was 29.5% (95% CI: 28.9%, 30.1%) (**Table 3**). The prevalence of non-vaccination was lower with lower levels of self-rated health. In relation to health behaviors, current daily smokers had a higher prevalence of non-vaccination (43.1%, 95% CI: 40.0%, 46.3%) than current non-smokers (28.8%, 95% CI: 28.2%, 29.4%). Nova Scotia residents had the lowest prevalence of non-vaccination (18.5%, 95% CI: 16.8%, 20.1%), while Quebec residents had the highest prevalence of non-vaccination (41.0%, 95% CI: 39.5%, 42.5%). All types of CMC had similar vaccination coverage.

**Table 3:** Factors associated with seasonal influenza non-vaccination among Canadian residents aged 65 years and older (N = 23,226).

	Proportion Unvaccinated among Each Response Category (95% CI)	Model 1^a^ N = 20,117	Model 2^a^ N = 20,065	Model 3^a^ N = 19,957
		aOR (95% CI)	aOR (95% CI)	aOR (95% CI)
Age (years)				
85–94	0.20 (0.19, 0.22)	Ref	Ref	Ref
75–84	0.23 (0.22, 0.24)	1.23 (1.06, 1.43)	1.21 (1.04, 1.41)	1.20 (1.04, 1.40)
65–74	0.35 (0.34, 0.36)	2.29 (1.98, 2.64)	2.16 (1.87, 2.50)	2.12 (1.82, 2.45)
Province of Residence				
Ontario	0.24 (0.23, 0.26)	Ref	Ref	Ref
Newfoundland	0.34 (0.32, 0.37)	1.62 (1.41, 1.86)	1.66 (1.44, 1.90)	1.63 (1.42, 1.88)
Prince Edward Island	0.24 (0.20, 0.28)	0.81 (0.64, 1.04)	0.78 (0.61, 0.99)	0.79 (0.62, 1.01)
Nova Scotia	0.19 (0.17, 0.20)	0.63 (0.54, 0.73)	0.63 (0.54, 0.73)	0.63 (0.55, 0.74)
New Brunswick	0.28 (0.25, 0.32)	1.00 (0.81, 1.25)	0.99 (0.80, 1.23)	0.97 (0.78, 1.21)
Quebec	0.41 (0.40, 0.43)	1.99 (1.80, 2.19)	1.93 (1.75, 2.14)	2.01 (1.82, 2.22)
Manitoba	0.29 (0.27, 0.31)	1.13 (1.00, 1.29)	1.13 (0.99, 1.29)	1.15 (1.01, 1.31)
Saskatchewan	0.31 (0.27, 0.35)	1.25 (1.00, 1.55)	1.24 (0.99, 1.54)	1.24 (1.00, 1.54)
Alberta	0.26 (0.25, 0.28)	1.12 (0.99, 1.27)	1.14 (1.01, 1.29)	1.15 (1.01, 1.30)
British Columbia	0.30 (0.29, 0.32)	1.33 (1.21, 1.47)	1.35 (1.22, 1.49)	1.37 (1.24, 1.51)
Sex				
Female	0.30 (0.29, 0.31)	Ref	Ref	Ref
Male	0.29 (0.28, 0.30)	1.10 (1.03, 1.18)	1.09 (1.02, 1.16)	1.10 (1.03, 1.18)
Urbanicity				
Urban	0.29 (0.28, 0.29)	Ref	Ref	Ref
Rural	0.34 (0.32, 0.36)	1.25 (1.14, 1.37)	1.22 (1.12, 1.34)	1.22 (1.11, 1.34)
Household Income (Canadian dollars)				
< 20000	0.41 (0.39, 0.44)	Ref	Ref	Ref
≥20000-<50000	0.34 (0.32, 0.35)	0.72 (0.63, 0.82)	0.74 (0.65, 0.85)	0.78 (0.67, 0.89)
≥50000-<100000	0.27 (0.26, 0.28)	0.49 (0.43, 0.56)	0.51 (0.44, 0.58)	0.55 (0.47, 0.64)
≥100000-<150000	0.24 (0.22, 0.25)	0.40 (0.34, 0.46)	0.41 (0.35, 0.48)	0.45 (0.38, 0.53)
≥150000	0.26 (0.23, 0.28)	0.45 (0.38, 0.54)	0.47 (0.39, 0.57)	0.51 (0.42, 0.62)
Education				
Less than secondary school graduation	0.33 (0.31, 0.35)	Ref	Ref	Ref
Secondary school graduation, no post-secondary education	0.32 (0.30, 0.34)	1.00 (0.87, 1.16)	1.01 (0.87, 1.17)	1.03 (0.89, 1.20)
Some post-secondary education	0.31 (0.29, 0.33)	1.02 (0.87, 1.20)	1.04 (0.88, 1.21)	1.05 (0.90, 1.24)
Post-secondary degree/diploma	0.29 (0.28, 0.29)	0.86 (0.76, 0.97)	0.88 (0.77, 0.99)	0.91 (0.80, 1.03)
Race				
White	0.29 (0.29, 0.30)	Ref	Ref	Ref
Non-White	0.36 (0.33, 0.40)	1.53 (1.30, 1.81)	1.54 (1.30, 1.82)	1.44 (1.22, 1.71)
CMC by Type				
Heart Disease	0.24 (0.22, 0.25)	0.80 (0.73, 0.88)	0.89 (0.80, 0.98)	0.89 (0.80, 1.00)
Respiratory Disorders	0.23 (0.21, 0.25)	0.69 (0.61, 0.79)	0.75 (0.66, 0.86)	0.73 (0.64, 0.84)
Kidney Disease or Failure	0.25 (0.22, 0.28)	0.85 (0.72, 1.00)	0.90 (0.76, 1.07)	0.89 (0.75, 1.06)
Asthma	0.23 (0.21, 0.24)	0.70 (0.63, 0.78)	0.76 (0.67, 0.86)	0.77 (0.68, 0.87)
Diabetes	0.25 (0.24, 0.26)	0.75 (0.69, 0.81)	0.82 (0.74, 0.91)	0.82 (0.74, 0.90)
Cancer	0.25 (0.24, 0.26)	0.80 (0.74, 0.86)	0.89 (0.80, 0.98)	0.90 (0.81, 0.99)
Other CMC	0.26 (0.26, 0.27)	0.78 (0.73, 0.84)	0.87 (0.78, 0.96)	0.87 (0.78, 0.97)
Number of CMC				
0	0.37 (0.36, 0.39)		Ref	Ref
1	0.31 (0.30, 0.33)		0.96 (0.86, 1.08)	0.96 (0.85, 1.08)
≥2	0.24 (0.23, 0.25)		0.86 (0.70, 1.05)	0.85 (0.69, 1.04)
Care or Assistance Received by Type				
Professional	0.23 (0.21, 0.25)		0.85 (0.76, 0.96)	0.84 (0.75, 0.95)
Non-Professional	0.25 (0.24, 0.27)		1.01 (0.92, 1.11)	1.00 (0.91, 1.10)
Healthcare Utilization by Type				
Family Doctor Contact	0.28 (0.27, 0.29)		0.47 (0.41, 0.54)	0.48 (0.42, 0.54)
Specialist Contact	0.26 (0.26, 0.27)		0.78 (0.73, 0.84)	0.79 (0.74, 0.85)
Hospitalization History	0.26 (0.25, 0.28)		1.06 (0.95, 1.19)	1.07 (0.95, 1.19)
Self-Rated Health				
Excellent	0.34 (0.32, 0.35)			Ref
Very Good	0.30 (0.29, 0.31)			0.91 (0.83, 1.00)
Good	0.28 (0.27, 0.29)			0.89 (0.80, 0.98)
Fair	0.27 (0.26, 0.29)			0.92 (0.80, 1.05)
Poor	0.23 (0.19, 0.26)			0.75 (0.58, 0.97)
Number in Household				
0	0.31 (0.30, 0.32)			Ref
1	0.28 (0.27, 0.29)			0.98 (0.90, 1.06)
≥2	0.34 (0.32, 0.36)			1.28 (1.13, 1.45)
Exercise				
None or Seldom	0.30 (0.29, 0.30)			Ref
Sometimes or Often	0.30 (0.28, 0.31)			0.98 (0.90, 1.07)
Current Smoking				
Not at All	0.29 (0.28, 0.29)			Ref
Occasionally	0.38 (0.31, 0.44)			1.14 (0.81, 1.60)
Daily	0.43 (0.40, 0.46)			1.55 (1.33, 1.81)
Alcohol				
Never	0.32 (0.30, 0.33)			Ref
Occasionally	0.33 (0.31, 0.34)			1.04 (0.92, 1.17)
Regular	0.28 (0.28, 0.29)			0.83 (0.76, 0.91)

^a^Grey cells indicate variables that were not included in the model represented by that column.

**Table 3** presents the results of the logistic regression analyses for adults aged 65 years and older. The results from the fully adjusted model did not differ substantively from the results of the partially adjusted models (models 1 and 2). We found in the fully adjusted model (model 3) that those aged 65–74, compared to those aged 85–94, had the highest odds ratio for being unvaccinated (aOR: 2.12, 95% CI: 1.82, 2.45) (**Table 3**). Those who were classified as non-white had higher odds of non-vaccination (1.44, 95% CI: 1.22, 1.71) than those who identified as white. Current daily smoking was also associated with higher odds of non-vaccination (1.55, 95% CI: 1.33, 1.81) compared to current non-smokers. Those who had 2 or more additional members in their household had higher odds of remaining unvaccinated (1.28, 95% CI: 1.13, 1.45) compared to those living alone. Residents of Quebec had the highest odds of non-vaccination by province (2.01, 95% CI: 1.82, 2.22), while Nova Scotia residents had notably lower odds of remaining unvaccinated (0.63, 95% CI: 0.55, 0.74). Those who reported contact with a family doctor in the past 12 months had markedly lower odds of remaining unvaccinated (0.48, 95% CI: 0.42, 0.54) compared to those without contact. Those who had contact with a medical specialist in the past 12 months had lower odds of remaining unvaccinated (0.79, 95% CI: 0.74, 0.85) than those without contact. Compared to those with excellent self-reported health, those with poor self-reported health had lower odds of remaining unvaccinated (0.75, 95% CI: 0.58, 0.97).

### 3.3 Influenza non-vaccination in adults aged 46–64 with at least one CMC

In our second group of interest, adults aged 46–64 with at least one CMC (N = 11,250) who are also routinely recommended to receive annual influenza vaccination, 50.4% (95% CI: 49.4%, 51.3%) of participants reported not receiving influenza vaccination in the past 12 months at the time of survey (**Table 4**). The percentage of participants who reported not being vaccinated decreased as age increased. Quebec residents had the highest prevalence of non-vaccination (66.9%, 95% CI: 64.9, 68.9%) among the provinces. A higher percentage of those who were current daily smokers reported non-vaccination than those who currently did not smoke (60.7%, 95% CI: 57.4%, 63.8% compared to 49.2%, 95% CI: 48.3%, 50.2%, respectively). Vaccination coverage did not notably differ across type of CMC.

**Table 4:** Factors associated with seasonal influenza non-vaccination among Canadian residents aged 46–64 years with at least one chronic medical condition (CMC) (N = 11,250).

	Proportion Unvaccinated among Each Response Category (95% CI)	Model 1^a^ N = 10,551	Model 2^a^ N = 10,522	Model 3^a^ N = 10,502
		aOR (95% CI)	aOR (95% CI)	aOR (95% CI)
Age (years)				
55–64	0.48 (0.47, 0.49)	Ref	Ref	Ref
46–54	0.57 (0.55, 0.59)	1.44 (1.31, 1.58)	1.43 (1.30, 1.57)	1.37 (1.25, 1.51)
Province of Residence				
Ontario	0.46 (0.44, 0.48)	Ref	Ref	Ref
Newfoundland	0.54 (0.50, 0.57)	1.34 (1.13, 1.58)	1.39 (1.17, 1.64)	1.39 (1.17, 1.65)
Prince Edward Island	0.46 (0.39, 0.54)	0.83 (0.60, 1.14)	0.80 (0.58, 1.11)	0.80 (0.58, 1.11)
Nova Scotia	0.35 (0.32, 0.38)	0.56 (0.47, 0.66)	0.57 (0.48, 0.67)	0.57 (0.48, 0.68)
New Brunswick	0.49 (0.43, 0.55)	1.03 (0.79, 1.33)	1.05 (0.80, 1.36)	1.09 (0.84, 1.43)
Quebec	0.67 (0.65, 0.69)	2.27 (2.00, 2.58)	2.23 (1.96, 2.53)	2.21 (1.95, 2.51)
Manitoba	0.49 (0.46, 0.52)	1.08 (0.93, 1.27)	1.09 (0.93, 1.27)	1.09 (0.93, 1.28)
Saskatchewan	0.50 (0.44, 0.56)	1.13 (0.86, 1.49)	1.13 (0.85, 1.50)	1.13 (0.85, 1.50)
Alberta	0.43 (0.40, 0.46)	0.84 (0.72, 0.97)	0.84 (0.72, 0.97)	0.84 (0.72, 0.97)
British Columbia	0.49 (0.47, 0.51)	1.09 (0.96, 1.23)	1.10 (0.97, 1.25)	1.12 (0.99, 1.26)
Sex				
Female	0.49 (0.47, 0.50)	Ref	Ref	Ref
Male	0.52 (0.51, 0.54)	1.22 (1.12, 1.32)	1.20 (1.10, 1.30)	1.18 (1.09, 1.28)
Urbanicity				
Urban	0.49 (0.48, 0.50)	Ref	Ref	Ref
Rural	0.56 (0.54, 0.58)	1.30 (1.16, 1.47)	1.28 (1.13, 1.44)	1.30 (1.15, 1.46)
Household Income (Canadian dollars)				
< 20000	0.52 (0.47, 0.56)	Ref	Ref	Ref
≥20000-<50000	0.54 (0.52, 0.57)	1.02 (0.83, 1.26)	1.00 (0.81, 1.24)	1.00 (0.81, 1.25)
≥50000-<100000	0.51 (0.50, 0.53)	0.90 (0.73, 1.09)	0.87 (0.71, 1.07)	0.88 (0.71, 1.08)
≥100000-<150000	0.51 (0.49, 0.53)	0.92 (0.75, 1.13)	0.90 (0.73, 1.11)	0.90 (0.71, 1.12)
≥150000	0.46 (0.44, 0.48)	0.71 (0.58, 0.88)	0.70 (0.56, 0.86)	0.69 (0.55, 0.87)
Education				
Less than secondary school graduation	0.58 (0.53, 0.62)	Ref	Ref	Ref
Secondary school graduation, no post-secondary education	0.53 (0.50, 0.56)	0.89 (0.69, 1.14)	0.89 (0.69, 1.14)	0.88 (0.68, 1.13)
Some post-secondary education	0.49 (0.46, 0.53)	0.87 (0.67, 1.13)	0.88 (0.68, 1.15)	0.87 (0.67, 1.13)
Post-secondary degree/diploma	0.50 (0.49, 0.51)	0.77 (0.62, 0.96)	0.88 (0.62, 0.98)	0.78 (0.62, 0.98)
Race				
White	0.50 (0.49, 0.51)	Ref	Ref	Ref
Non-White	0.53 (0.49, 0.56)	1.16 (0.98, 1.38)	1.16 (0.98, 1.38)	1.15 (0.96, 1.36)
CMC by Type				
Heart Disease	0.47 (0.44, 0.50)	0.77 (0.68, 0.89)	0.90 (0.77, 1.04)	0.91 (0.78, 1.06)
Respiratory Disorders	0.45 (0.42, 0.48)	0.76 (0.66, 0.88)	0.87 (0.74, 1.02)	0.84 (0.72, 0.99)
Kidney Disease or Failure	0.49 (0.45, 0.54)	0.79 (0.65, 0.98)	0.91 (0.73, 1.12)	0.92 (0.74, 1.14)
Asthma	0.47 (0.45, 0.48)	0.69 (0.63, 0.76)	0.80 (0.70, 0.90)	0.81 (0.71, 0.92)
Diabetes	0.48 (0.46, 0.49)	0.79 (0.72, 0.87)	0.91 (0.80, 1.03)	0.93 (0.82, 1.05)
Cancer	0.48 (0.46, 0.50)	0.82 (0.73, 0.91)	0.98 (0.85, 1.12)	0.98 (0.86, 1.13)
Other CMC	0.49 (0.48, 0.50)	0.74 (0.67, 0.80)	0.86 (0.76, 0.98)	0.87 (0.77, 0.99)
Number of CMC				
≥ 2	0.44 (0.43, 0.46)		Ref	Ref
1	0.55 (0.53, 0.56)		1.21 (1.04, 1.42)	1.23 (1.05, 1.43)
Care or Assistance Received by Type				
Professional	0.42 (0.38, 0.47)		0.79 (0.64, 0.96)	0.81 (0.66, 0.99)
Non-Professional	0.45 (0.43, 0.48)		0.92 (0.81, 1.04)	0.93 (0.82, 1.06)
Healthcare Utilization by Type				
Family Doctor Contact	0.49 (0.48, 0.50)		0.53 (0.45, 0.62)	0.53 (0.45, 0.62)
Specialist Contact	0.47 (0.46, 0.48)		0.77 (0.70, 0.84)	0.78 (0.72, 0.85)
Hospitalization History	0.48 (0.45, 0.51)		1.11 (0.95, 1.30)	1.12 (0.96, 1.31)
Self-Rated Health				
Excellent	0.51 (0.49, 0.54)			Ref
Very Good	0.52 (0.50, 0.53)			1.06 (0.93, 1.20)
Good	0.51 (0.49, 0.52)			1.03 (0.89, 1.18)
Fair	0.46 (0.43, 0.49)			0.92 (0.77, 1.10)
Poor	0.43 (0.37, 0.48)			0.80 (0.60, 1.07)
Number in Household				
0	0.51 (0.49, 0.54)			Ref
1	0.48 (0.47, 0.50)			0.95 (0.84, 1.08)
≥ 2	0.53 (0.51, 0.55)			1.13 (0.98, 1.29)
Exercise				
None or Seldom	0.51 (0.50, 0.52)			Ref
Sometimes or Often	0.49 (0.48, 0.51)			0.95 (0.86, 1.04)
Current Smoking				
Not at All	0.49 (0.48, 0.50)			Ref
Occasionally	0.59 (0.52, 0.65)			1.22 (0.92, 1.63)
Daily	0.61 (0.57, 0.64)			1.56 (1.33, 1.83)
Alcohol				
Never	0.49 (0.46, 0.51)			Ref
Occasionally	0.47 (0.45, 0.50)			0.94 (0.80, 1.11)
Regular	0.51 (0.50, 0.52)			1.07 (0.93, 1.22)

^a^Grey cells indicate variables that were not included in the model represented by that column.

**Table 4** presents the results of the logistic regression analysis for adults aged 46–64 years with at least one CMC. The results remained consistent across the partially adjusted models (models 1 and 2) and the fully adjusted model (model 3). For our full model, the odds of remaining unvaccinated were highest for Quebec residents (2.21, 95% CI: 1.95, 2.51), while Nova Scotia residents had the lowest odds of remaining unvaccinated (0.57, 95% CI: 0.48, 0.68), compared to Ontario residents after controlling for all other variables (**Table 4**). Current daily smokers had higher odds of non-vaccination than current non-smokers (1.56, 95% CI: 1.33, 1.83). Those aged 46–54 had higher odds of non-vaccination than those aged 55–64 (1.37, 95% CI: 1.25, 1.51). Residence in rural areas was associated with higher odds of non-vaccination (1.30, 95% CI: 1.15, 1.46) compared to residence in urban areas. No type of CMC was notably associated with lower odds of remaining unvaccinated, although those with 1 CMC had higher odds of non-vaccination than those with 2 or more CMC (1.23, 95% CI: 1.05, 1.43). Contact with a family doctor in the past 12 months was associated with notably low odds of remaining unvaccinated (0.53, 95% CI: 0.45, 0.62), as was specialist contact in the past 12 months (0.78, 95% CI: 0.72, 0.85).

### 3.4 Sensitivity analysis

We found no evidence to suggest that there were significant differences in self-reported influenza vaccine uptake according to whether or not a participant was asked to report during or after the typical flu season or by cohort for those aged 65 years and older (Comprehensive cohort, p-value = 0.49; Tracking cohort, p-value = 0.23) and those aged 46–64 with at least one CMC (Comprehensive cohort, p-value = 0.74; Tracking cohort, p-value = 0.59).

## 4. Discussion

Despite the provision of publicly funded influenza vaccines for groups at high risk of severe outcomes across Canada and national recommendations for annual vaccination among these groups, annual estimates of influenza vaccination coverage remain below the target of 80% coverage set in 2005 [3, 4]. We found that the prevalence of non-vaccination was 29.5% (95% CI: 28.9%, 30.1%) among those aged 65 and older and 50.4% (95% CI: 49.4%, 51.3%) among those aged 46–64 who had at least one CMC over 2015–2018. In addition, the prevalence of non-vaccination varied widely by province. In this study, we describe the prevalence of influenza non-vaccination and identify factors associated with non-vaccination using the large and robust data available from the CLSA. In the fully adjusted model, for both groups, contact with a family doctor or specialist within the last 12 months compared to those without contact and those who reported current non-smoking compared to those who reported smoking were less likely to be unvaccinated.

Many characteristics have been associated with missed influenza vaccination in a given season among Canadian adults. These include sociodemographic characteristics (such as younger age, lower education level, and residence in Quebec compared to other provinces), health-related factors (such as smoking, increasing self-rated health, and absence of CMCs), and lack of healthcare utilization [26, 33–35]. However, most studies have focused on a limited set of covariates and did not account for the extensive range of characteristics that have been associated with vaccination in other settings or are of general public health importance, so it is difficult to assess the relative importance of multiple factors simultaneously. Smaller studies have had insufficient power to adequately assess differences or may be subject to bias due to their limited response rates or other factors. At the national level, influenza vaccination coverage in Canada is primarily assessed via the annual Seasonal Influenza Vaccination Coverage Survey [36]. However, additional data are needed to complement these estimates as these coverage surveys tend to have response rates around 20%, only include about 2,000 adults each year, and are not designed to assess the broad range of characteristics that may be associated with non-vaccination [3, 13, 14]. The vaccination coverage estimates in our study were similar to those reported by these surveys during the period of CLSA data collection (2015–2018) [17], although our study captures a greater depth of information on important vaccination-associated factors, particularly healthcare utilization and self-rated health. These additional variables can help specify who among these high-risk groups is not getting the recommended vaccine and assist with the identification of new targets for vaccination policies. Additionally, our use of nested models allowed us to investigate the independent association between a complex set of covariates and the outcome while controlling for the associations of an increasingly comprehensive set of other variables.

Differences in existing policies, vaccination programs, vaccination barriers, and awareness locally may contribute to the variations in influenza vaccine uptake by province that were observed in this study. Most provinces and territories in Canada had a universal recommendation for influenza vaccination for everyone aged 6 months and up at the time of data collection [8]. Notably, both Quebec and British Columbia did not have a universal recommendation [8, 34] at that time and here had a lower prevalence of influenza vaccination among groups that would be particularly recommended for influenza vaccination compared to other provinces. Overall, provinces with lower coverage may require additional efforts to increase influenza vaccination uptake.

One of the key opportunities for increasing awareness of influenza vaccination recommendations comes through healthcare encounters; therefore, it is important to maximize the potential for encouraging vaccination during these visits. We found that those who had visited a medical specialist, or, particularly strongly, a family doctor in the past 12 months were more likely to report having been vaccinated among both high-risk groups. Similarly, data from the 2003 CCHS found that among participants (aged 12 years and older) with self-reported asthma and chronic obstructive pulmonary disease, those without a family doctor were less likely to have received influenza vaccination within the past year [37]. However, another analysis of these data found that hospital admission was associated with a greater proportion of influenza vaccination in the past year [33], which was not a factor identified in our analysis.

The association of influenza non-vaccination with healthcare utilization may be related to the participant being exposed to vaccination recommendations from health providers. For example, a survey of subspecialists in the United States who provide outpatient care to patients aged 18–64 at high risk of severe outcomes of influenza found that stocking and recommendation of the influenza vaccine varies [38, 39]. In another Canadian study of influenza vaccination, researchers reported that presence of a CMC was associated with lower odds of non-vaccination, possibly due to increased contact with health professionals [34]. A systematic review of methods of influenza vaccination distribution in Australia, Canada, England, Finland, the Netherlands, New Zealand, Sweden, Switzerland, and the United States, found that influenza coverage could be increased in healthcare settings through methods such as standing orders for influenza vaccination in hospital and tertiary-care settings [40]. Another systematic review found that use of Personal Electronic Health Records (PEHR) in the United States and Australia can improve influenza vaccine uptake [41]. Although the use of PEHR in Canada is variable, it is expanding [42], and the use of PEHR may become an increasingly important tool for prompting physician vaccine recommendations. These findings could be adapted to address the lack of vaccination in those aged 46–64 with at least one CMC and a history of hospitalization, in particular.

On the other hand, we also found that people who were healthier, or who had better self-rated health, were less likely to have received the influenza vaccine. These individuals are also likely to have had fewer healthcare encounters in the previous year. Expanded access to pharmacy-based clinics has made influenza vaccinations widely available, and these could be a source of increased opportunity for vaccination in under-vaccinated groups with good self-rated health who may not see the need for influenza vaccination [43]. It may be that enhancing opportunities for personalized messaging in healthcare encounters and for public health messaging emphasizing the importance of preventing influenza and its complications for healthy older adults and younger adults with CMC who do not view themselves as vulnerable to influenza could be specifically beneficial to disseminate information about influenza vaccine safety, efficacy, and benefits in groups at high risk of severe outcomes.

Our study provides additional evidence that builds upon previous findings about factors associated with influenza vaccination among older adults. Consistent with some previous studies [33, 38, 44, 45], we found a significant association between poor self-rated health and non-vaccination for those aged 65 years and older, after adjusting for other relevant factors. Although outside the scope of our current study, future studies could also examine the relationship between frailty, which is not typically measured or captured under other health status indicators such as CMC, and vaccination status in order to determine whether it is associated vaccination uptake.

Lastly, the COVID-19 pandemic changed the influenza landscape and demonstrated the ongoing the importance of routine vaccination. During the 2020/2021 influenza season, Canada reported no community circulation of influenza [46], and a markedly lower number of influenza cases were reported compared to previous seasons despite an increased number of influenza tests performed [47]. Despite this initial decrease in cases during the SARS-CoV-2 pandemic, in the 2021/2022 Canadian season, influenza activity and related healthcare visits rose [46, 48], and the vaccination coverage of groups at high risk of severe outcomes in 2020–2021 was similar to that of the prior influenza season [46]. As influenza seasons during the SARS-CoV-2 pandemic begin to regain their typical severity and frequency of infection and as influenza vaccination has plateaued in groups at high risk of severe outcomes, it remains critical to continue investigating patterns of influenza vaccination uptake, especially among groups at high risk of severe outcomes. Capitalizing on COVID-19 vaccine awareness may also be a method to improve influenza vaccination rates: annual (and potentially combined) seasonal boosters for COVID-19 and influenza could encourage both vaccinations in those who otherwise would not get vaccinated.

Vaccination decisions are complex and multiple factors contribute to observed vaccination rates and trends over time. As suggested by the framework the World Health Organization proposed in their behavioral and social drivers (BeSD) model, attitudes towards vaccination, social structures, self-motivation, and practical issues such as access to vaccines all contribute to individual-level decisions about receiving a vaccine [49]. Our analysis focuses on characterizing and describing vaccine uptake among high-risk populations only given that data about the individual, societal, and structural factors that led to non-vaccination were not collected in the context of this study. While understanding patterns of uptake is an important first step, future studies should be designed to investigate the complex factors that shape vaccine decisions and how they interact to lead to the trends in vaccination observed.

This study has multiple strengths, including the ability to precisely estimate vaccination coverage among multiple groups due to the large sample size, the provision of province-level vaccination estimates, and the ability to assess a wide range of covariates across many vaccination-associated domains that include novel factors not examined in other studies. This study, as with most observational studies, has several limitations. For one, our results apply only to community-dwelling adults since participants living in institutions were excluded at the CLSA baseline [50]. The CLSA over-represents those with higher education and socioeconomic status compared to the general population [18], which may influence vaccination knowledge, attitudes, beliefs, and behaviors. Additionally, many of the variables used in this study are based on self-report, which may be more prone to bias compared to objective measures; however, the key outcome of interest, self-reported influenza vaccination, has been validated in previous studies [23, 24]. It is also possible that the missing data and listwise deletion that occurred in the regression models may not be missing entirely at random, which could bias our results if the missingness was informative. Lastly, the datasets used in this study did not include information on participants’ reasons for not receiving an influenza vaccine in the past 12 months; further research is needed to characterize why participants did not receive a recommended influenza vaccine despite being eligible, to understand the relative importance of these factors as drivers of non-vaccination, and to assess the impact of modifying these factors on uptake.

## 5. Conclusion

Influenza vaccination coverage from 2015–2018 among individuals at high risk of severe influenza remained below national targets in Canada, particularly among those aged 46–64 years with at least one CMC but also among those aged 65 years and older. The high prevalence of non-vaccination demonstrates the need to substantially increase vaccination uptake to meet the 80% coverage target by 2025. For both groups, recent contact with a family doctor was strongly associated with being less likely to remain unvaccinated. This suggests that interventions to increase influenza vaccination among groups at high risk of severe outcomes should evaluate the effectiveness of increasing awareness about vaccination recommendations among the general population especially in venues that older adults and those with CMC frequent outside of healthcare settings to improve overall vaccination rates, particularly in provinces where coverage is lower.

## Supporting information

S1 Table CLSA survey questions, variables, responses options, and variable categorization. (PDF)

S1 File (DOCX)
