# Supplementary material for: Influenza vaccine coverage and factors associated with non-vaccination among adults at high risk for severe outcomes: An analysis of the Canadian Longitudinal Study on Aging
Source: PLoS One. 2022 Sep 30;17(9):e0275135. doi: 10.1371/journal.pone.0275135 (PMC9524702; doi:10.1371/journal.pone.0275135)
Supplement: S1 Table — (PDF) [file pone.0275135.s001.pdf]

Supplementary Table 1: CLSA survey questions, variables, responses options, and variable categorization

CLSA Survey Question and Variable Label	CLSA Response Options → Variable Categorization for Analysis
Have you had… Flu shot in the last 12 months<br>PHB_FLUV_COF1[a]<br>PHB_FLUV_TRF1[a]	1 = Yes<br>2 = No<br>9 = Refused → NA<br>-88888 = Missing → NA
Participant age at FU1 (in years)<br>AGE_NMBR_COF1<br>AGE_NMBR_TRF1	46-54, 55-64, 65-74, 75-84, 85-94
Province of residence<br>WGHTS_PROV_COF1<br>WGHTS_PROV_TRF1	Newfoundland, Prince Edward Island[b], Nova Scotia, New Brunswick[b], Quebec, Ontario, Manitoba, Saskatchewan[b], Alberta, British Columbia
What was your sex at birth?<br>SDC_BTHSEX_COF1<br>SDC_BTHSEX_TRF1	1 = Male → 1<br>2 = Female → 0<br>8 = Don't know/No answer → NA<br>9 = Refused → NA<br>-88888 = Missing → NA
Urban/ rural classification<br>SDC_URBAN_RURAL_COF1<br>SDC_URBAN_RURAL_TRF1	0 = Rural area → Rural<br>1 = Urban core → Urban<br>2 = Urban fringe → Urban<br>3 = Rural fringe in CMA/CAs → Rural<br>4 = Urban Areas out CMA/CAs → Urban<br>5 = Rural fringe out CMA/CAs → Rural<br>6 = Secondary urban core → Urban<br>9 = Link to DA → Rural<br>-88888 = Missing → NA
What is your best estimate of the total household income received by all household members, from all sources, before taxes and deductions, in the past 12 months?<br>INC_TOT_COF1<br>INC_TOT_TRF1	1 = Less than $20,000<br>2 = $20,000 or more, but less than $50,000<br>3 = $50,000 or more, but less than $100,000<br>4 = $100,000 or more, but less than $150,000<br>5 = $150,000 or more<br>8 = Don't know/No answer → NA<br>9 = Refused → NA<br>-88888 = Missing → NA
Highest Level of Education - Respondent, 4 Levels<br>ED_UDR04_COM<br>ED_UDR04_TRM	1 = Less than secondary school graduation<br>2 = Secondary school graduation, no post-secondary education<br>3 = Some post-secondary education<br>4 = Post-secondary degree/diploma<br>9 = At least one required question was not answered → NA
Cultural / Racial Background<br>SDC_DCGT_COM<br>SDC_DCGT_TRM	1 = White only → White<br>2 = Black only → Non-White<br>3 = Korean only → Non-White<br>4 = Filipino only → Non-White<br>5 = Japanese only → Non-White<br>6 = Chinese only → Non-White<br>7 = South Asian only → Non-White<br>8 = Southeast Asian only → Non-White<br>9 = Arab only → Non-White<br>10 = West Asian only → Non-White<br>11 = Latin American only → Non-White<br>12 = Other racial or cultural origin (only) → Non-White<br>13 = Multiple racial or cultural origins → Non-White

	99 = Required question was not answered → NA
**Type of CMC**<br>Has a doctor ever told you that you have heart disease (including congestive heart failure or CHF)?<br>CCC_HEART_COF1<br>CCT_HEART_TRF1	1 = Yes<br>2 = No<br>8 = Don't know/No answer → NA<br>9 = Refused → NA<br>-88880 = Did not complete a DCS visit → NA<br>-88888 = Missing → NA
**Type of CMC**<br>Has a doctor told you that you have/had any of the following: emphysema, chronic bronchitis, chronic obstructive pulmonary disease (COPD), or chronic changes in lungs due to smoking?<br>CCC_COPD_COF1<br>CCT_COPD_TRF1	1 = Yes<br>2 = No<br>8 = Don't know/No answer → NA<br>9 = Refused → NA<br>-88880 = Did not complete a DCS visit → NA<br>-88888 = Missing → NA
**Type of CMC**<br>Has a doctor ever told you that you have kidney disease or kidney failure?<br>CCC_KIDN_COF1<br>CCT_KIDN_TRF1	1 = Yes<br>2 = No<br>8 = Don't know/No answer → NA<br>9 = Refused → NA<br>-88880 = Did not complete a DCS visit → NA<br>-88888 = Missing → NA
**Type of CMC**<br>Has a doctor ever told you that you have asthma?<br>CCC_ASTHM_COF1<br>CCT_ASTHM_TRF1	1 = Yes<br>2 = No<br>8 = Don't know/No answer → NA<br>9 = Refused → NA<br>-88880 = Did not complete a DCS visit → NA<br>-88888 = Missing → NA
**Type of CMC**<br>Has a doctor ever told you that you have diabetes, borderline diabetes or that your blood sugar is high?<br>DIA_DIAB_COF1<br>CCT_DIAB_TRF1	1 = Yes<br>2 = No<br>8 = Don't know/No answer → NA<br>9 = Refused → NA<br>-88888 = Missing → NA<br>-88880 = Did not complete a DCS visit → NA
**Type of CMC**<br>Has a doctor ever told you that you had cancer?<br>CCC_CANC_COF1<br>CCT_CANC_TRF1	1 = Yes<br>2 = No<br>8 = Don't know/No answer → NA<br>9 = Refused → NA<br>-88880 = Did not complete a DCS visit → NA<br>-88888 = Missing → NA
**Type of CMC**<br>Other CMC: **composite variable**[c]<br><br>Has a doctor ever told you that you have had a heart attack or myocardial infarction?;<br>Has a doctor ever told you that you have high blood pressure or hypertension?; Has a doctor ever told you that you have dementia or Alzheimer's disease?;. Has a doctor ever told you that you had Parkinsonism or Parkinson's Disease?; Has a doctor ever told you that you have experienced a Stroke or CVA (cerebrovascular accident)?; Has a doctor ever told you that you have experienced a ministroke or TIA (Transient Ischemic Attack)?<br>CCC_AMI_COF1 and/or CCC_HBP_COF1 and/or CCC_ALZH_COF1 and/or CCC_PARK_COF1	0 = No other CMC<br>1 = Other CMC

and/or CCC_CVA_COF1 and/or CCC_TIA_COF1 and/or<br>CCT_AMI_TRF1 and/or CCT_HBP_TRF1 and/or CCT_ALZH_TRF1 and/or PKD_PARK_TRF1 and/or CCT_CVA_TRF1 and/or CCT_TIA_TRF1	
Number of CMC: **calculated variable**[d]<br><br>Heart disease (Y = 1, N = 0) + lung problem (Y = 1, N = 0) + kidney disease (Y = 1, N = 0) + asthma (Y = 1, N = 0) + diabetes (Y = 1, N = 0) + cancer (Y = 1, N = 0) + other CMC (Y = 1, N = 0) = ∑Y = Total CMC	0 = 0<br>1 = 1<br>2-7 = ≥2
Received professional care in past 12 months: **composite variable**[c]<br><br><u>During the past 12 months, did you receive short-term or long-term professional assistance at home, because of a health condition or limitation that affects your daily life, for any of the following activities?:</u><br><br><u>Received professional personal care;</u><br><u>Received professional medical care;</u><br><u>Received professional managing care;</u><br><u>Received professional assistance with meal preparation or delivery; Received professional assistance with activities;</u><br><u>Received professional assistance with transportation;</u><br><u>Received professional physical therapy;</u><br><u>Received professional training and adaptation assistance;</u><br><u>Received other professional assistance</u><br>CR1_PRO_PR_COF1 and/or CR1_PRO_MD_COF1 and/or CR1_PRO_MG_COF1 and/or CR1_PRO_MH_COF1 and/or CR1_PRO_WK_COF1 and/or CR1_PRO_TR_COF1 and/or CR1_PRO_PT_COF1 and/or CR1_PRO_TA_COF1 and/or CR1_PRO_OT_COF1 and/or CR1_PRO_PR_TRF1 and/or CR1_PRO_MD_TRF1 and/or CR1_PRO_MG_TRF1 and/or CR1_PRO_MH_TRF1 and/or CR1_PRO_WK_TRF1 and/or CR1_PRO_TR_TRF1 and/or CR1_PRO_PT_TRF1 and/or CR1_PRO_TA_TRF1 and/or CR1_PRO_OT_TRF1	0 = 0 → No professional care received for any activity listed<br>1 = 1 → Professional care received for any activity listed
Received non-professional care in past 12 months: **composite variable**[c]<br><br><u>Received non-professional personal care;</u><br><u>Received non-professional medical care;</u>	0 = 0 → No non-professional care received for any activity listed<br>1 = 1 → Non-professional care received for any activity listed

Received non-professional managing care;<br>Received non-professional assistance with activities;<br>Received non-professional assistance with transportation;<br>Received non-professional assistance with meal preparation;<br>Received non-professional physical therapy;<br>Received non-professional training and adaptation assistance;<br>Received other non-professional assistance<br>CR2_FAM_PR_COF1 and/or<br>CR2_FAM_MD_COF1 and/or<br>CR2_FAM_MG_COF1 and/or<br>CR2_FAM_WK_COF1 and/or<br>CR2_FAM_TR_COF1 and/or<br>CR2_FAM_MH_COF1 and/or<br>CR2_FAM_PT_COF1 and/or<br>CR2_FAM_TA_COF1 and/or<br>CR2_FAM_OT_COF1 and/or<br>CR2_FAM_PR_TRF1 and/or<br>CR2_FAM_MD_TRF1 and/or<br>CR2_FAM_MG_TRF1 and/or<br>CR2_FAM_WK_TRF1 and/or<br>CR2_FAM_TR_TRF1 and/or<br>CR2_FAM_MH_TRF1 and/or<br>CR2_FAM_PT_TRF1 and/or<br>CR2_FAM_TA_TRF1 and/or<br>CR2_FAM_OT_TRF1	
During the past 12 months, have you had contact with any of the following about your physical or mental health?<br>Has had contact with: Family Doctor<br>HCU_FAMPHY_COF1<br>HCU_FAMPHY_TRF1	1 = Yes<br>2 = No<br>8 = Don't know/No answer → NA<br>9 = Refused → NA<br>-88888 = Missing → NA
Has had contact with: Medical specialist (such as a Cardiologist, Gynaecologist, Psychiatrist or Ophthalmologist)<br>HCU_SPEC_COF1<br>HCU_SPEC_TRF1	1 = Yes<br>2 = No<br>8 = Don't know/No answer → NA<br>9 = Refused → NA<br>-88888 = Missing → NA
Were you a patient in a hospital overnight during the past 12 months?<br>HCU_HLOVRNT_COF1<br>HCU_HLOVRNT_TRF1	1 = Yes<br>2 = No<br>8 = Don't know/No answer → NA<br>9 = Refused → NA<br>-88888 = Missing → NA
In general, would you say your health is excellent, very good, good, fair, or poor?<br>GEN_HLTH_COF1<br>GEN_HLTH_TRF1	1 = Excellent<br>2 = Very good<br>3 = Good<br>4 = Fair<br>5 = Poor<br>8 = Don't know/No answer → NA<br>9 = Refused → NA<br>-88888 = Missing → NA
How many people, not including yourself, currently live in your household?: c**alculated variable**[d]<br>SN_LIVH_NB_COF1	0 = 0<br>1 = 1<br>2-9 = ≥2

SN_LIVH_NB_TRF1	-88880 = Did not complete a DCS visit → NA<br>-88888 = Missing → NA
Over the past 7 days, how often did you engage in moderate sports or recreational activities such as ballroom dancing, hunting, skating, golf without a cart, softball or other similar activities?; Over the past 7 days, how often did you engage in strenuous sports or recreational activities such as jogging, swimming, snowshoeing, cycling, aerobics, skiing or other similar activities?: **composite variable**[c]<br>PA2_MSPRT_COF1 and/or PA2_SSPRT_COF1 and/or<br>PA2_MSPRT_TRF1 and/or PA2_SSPRT_TRF1	1 = Never → None or Seldom<br>2 = Seldom (1 to 2 days) → None or Seldom<br>3 = Sometimes (3 to 4 days) → Sometimes or Often<br>4 = Often (5 to 7 days) → Sometimes or Often<br>8 = Don't know/No answer → NA<br>9 = Refused → NA<br>-88888 = Missing → NA
At the present time, do you smoke cigarettes daily, occasionally or not at all?<br>SMK_CURRCG_COF1<br>SMK_CURRCG_TRF1	1 = Daily (at least one cigarette every day for the past 30 days)<br>2 = Occasionally (at least one cigarette in the past 30 days, but not every day)<br>3 = Not at all (you did not smoke at all in the past 30 days)<br>8 = Don't know/No answer → NA<br>9 = Refused → NA<br>-88888 = Missing → NA
Type of Drinker (Past 12 Months)<br>ALC_TTM_COF1<br>ALC_TTM_TRF1	1 = Regular drinker (at least once a month)<br>2 = Occasional drinker<br>3 = Did not drink in the last 12 months<br>-77771 = Inconclusive due to at least one missing item → NA

[a]*COF1 indicates the Comprehensive cohort; TRF1 indicates the Tracking cohort*
[b]*There were no participants in the Comprehensive cohort in these provinces*
[c]*Respondents who responded "Y" or "1" to at least one of the multiple included variables were coded as "Y" or "sometimes or often" in the new composite variable*
[d]*Re-categorization of existing categories for single variable*
