# Supplementary material for: Influenza vaccine coverage and factors associated with non-vaccination among adults at high risk for severe outcomes: An analysis of the Canadian Longitudinal Study on Aging
Source: PLoS One. 2022 Sep 30;17(9):e0275135. doi: 10.1371/journal.pone.0275135 (PMC9524702; doi:10.1371/journal.pone.0275135)
Supplement: S1 File — (DOCX) [file pone.0275135.s002.docx]

**Table 1. Regression Analysis Output: Canadian residents aged 65 years and older (N=23,226)**

|  | Model 1^a^  N=20,117 | | | Model 2^a^  N=20,065 |  |  | Model 3^a^  N=19,957 | | |
| --- | --- | --- | --- | --- | --- | --- | --- | --- | --- |
|  | aOR  (95% CI) | p-value | Standard Error | aOR  (95% CI) | p-value | Standard Error | aOR  (95% CI) | p-value | Standard Error |
| Age (years) |  |  |  |  |  |  |  |  |  |
| 85-94 | Ref | Ref | Ref | Ref | Ref | Ref | Ref | Ref | Ref |
| 75-84 | 1.23 (1.06, 1.43) | 0.01 | 0.08 | 1.21 (1.04, 1.41) | 0.01 | 0.08 | 1.20 (1.04, 1.40) | 0.02 | 0.08 |
| 65-74 | 2.29 (1.98, 2.64) | < 2e-16 | 0.07 | 2.16 (1.87, 2.50) | < 2e-16 | 0.07 | 2.12 (1.82, 2.45) | < 2e-16 | 0.08 |
| Province of Residence |  |  |  |  |  |  |  |  |  |
| Ontario | Ref | Ref | Ref | Ref | Ref | Ref | Ref | Ref | Ref |
| Newfoundland | 1.62 (1.41, 1.86) | 1.42e-11 | 0.07 | 1.66 (1.44, 1.90) | 2.06e-12 | 0.07 | 1.63 (1.42, 1.88) | 1.44e-11 | 0.07 |
| Prince Edward Island | 0.81 (0.64, 1.04) | 0.1 | 0.12 | 0.78 (0.61, 0.99) | 0.04 | 0.13 | 0.79 (0.62, 1.01) | 0.06 | 0.13 |
| Nova Scotia | 0.63 (0.54, 0.73) | 9.22e-10 | 0.08 | 0.63 (0.54, 0.73) | 1.69e-09 | 0.08 | 0.63 (0.55, 0.74) | 3.48e-09 | 0.08 |
| New Brunswick | 1.00 (0.81, 1.25) | 0.98 | 0.11 | 0.99 (0.80, 1.23) | 0.93 | 0.11 | 0.97 (0.78, 1.21) | 0.82 | 0.11 |
| Quebec | 1.99 (1.80, 2.19) | < 2e-16 | 0.05 | 1.93 (1.75, 2.14) | < 2e-16 | 0.05 | 2.01 (1.82, 2.22) | < 2e-16 | 0.05 |
| Manitoba | 1.13 (1.00, 1.29) | 0.06 | 0.07 | 1.13 (0.99, 1.29) | 0.06 | 0.07 | 1.15 (1.01, 1.31) | 0.04 | 0.07 |
| Saskatchewan | 1.25 (1.00, 1.55) | 0.05 | 0.11 | 1.24 (0.99, 1.54) | 0.06 | 0.11 | 1.24 (1.00, 1.54) | 0.05 | 0.11 |
| Alberta | 1.12 (0.99, 1.27) | 0.07 | 0.06 | 1.14 (1.01, 1.29) | 0.04 | 0.06 | 1.15 (1.01, 1.30) | 0.03 | 0.06 |
| British Columbia | 1.33 (1.21, 1.47) | 1.30e-08 | 0.05 | 1.35 (1.22, 1.49) | 4.14e-09 | 0.05 | 1.37 (1.24, 1.51) | 1.25e-09 | 0.05 |
| Sex |  |  |  |  |  |  |  |  |  |
| Female | Ref | Ref | Ref | Ref | Ref | Ref | Ref | Ref | Ref |
| Male | 1.10 (1.03, 1.18) | 3.4e-03 | 0.03 | 1.09 (1.02, 1.16) | 0.01 | 0.03 | 1.10 (1.03, 1.18) | 4.9e-3 | 0.04 |
| Urbanicity |  |  |  |  |  |  |  |  |  |
| Urban | Ref | Ref | Ref | Ref | Ref | Ref | Ref | Ref | Ref |
| Rural | 1.25 (1.14, 1.37) | 1.08e-06 | 0.05 | 1.22 (1.12, 1.34) | 1.52e-05 | 0.05 | 1.22 (1.11, 1.34) | 1.92e-05 | 0.05 |
| Household Income (Canadian dollars) |  |  |  |  |  |  |  |  |  |
| < 20000 | Ref | Ref | Ref | Ref | Ref | Ref | Ref | Ref | Ref |
| ≥20000-<50000 | 0.72 (0.63, 0.82) | 1.64e-06 | 0.07 | 0.74 (0.65, 0.85) | 1.24e-05 | 0.07 | 0.78 (0.67, 0.89) | 4e-04 | 0.07 |
| ≥50000-<100000 | 0.49 (0.43, 0.56) | < 2e-16 | 0.07 | 0.51 (0.44, 0.58) | < 2e-16 | 0.07 | 0.55 (0.47, 0.64) | 1.94e-15 | 0.08 |
| ≥100000-<150000 | 0.40 (0.34, 0.46) | < 2e-16 | 0.08 | 0.41 (0.35, 0.48) | < 2e-16 | 0.08 | 0.45 (0.38, 0.53) | < 2e-16 | 0.09 |
| ≥150000 | 0.45 (0.38, 0.54) | < 2e-16 | 0.09 | 0.47 (0.39, 0.57) | 1.05e-15 | 0.09 | 0.51 (0.42, 0.62) | 1.43e-11 | 0.10 |
| Education |  |  |  |  |  |  |  |  |  |
| Less than secondary school graduation | Ref | Ref | Ref | Ref | Ref | Ref | Ref | Ref | Ref |
| Secondary school graduation, no post-secondary education | 1.00 (0.87, 1.16) | 0.96 | 0.07 | 1.01 (0.87, 1.17) | 0.89 | 0.07 | 1.03 (0.89, 1.20) | 0.65 | 0.07 |
| Some post-secondary education | 1.02 (0.87, 1.20) | 0.76 | 0.08 | 1.04 (0.88, 1.21) | 0.67 | 0.08 | 1.05 (0.90, 1.24) | 0.51 | 0.08 |
| Post-secondary degree/diploma | 0.86 (0.76, 0.97) | 0.01 | 0.06 | 0.88 (0.77, 0.99) | 0.03 | 0.06 | 0.91 (0.80, 1.03) | 0.12 | 0.06 |
| Race |  |  |  |  |  |  |  |  |  |
| White | Ref | Ref | Ref | Ref | Ref | Ref | Ref | Ref | Ref |
| Non-White | 1.53 (1.30, 1.81) | 3.31e-07 | 0.08 | 1.54 (1.30, 1.82) | 3.84e-07 | 0.08 | 1.44 (1.22, 1.71) | 1.95e-05 | 0.09 |
| CMC by Type |  |  |  |  |  |  |  |  |  |
| Heart Disease | 0.80 (0.73, 0.88) | 1.12e-06 | 0.05 | 0.89 (0.80, 0.98) | 0.02 | 0.05 | 0.89 (0.80, 1.00) | 0.04 | 0.05 |
| Lung Problems | 0.69 (0.61, 0.79) | 1.04e-08 | 0.06 | 0.75 (0.66, 0.86) | 3.18e-05 | 0.07 | 0.73 (0.64, 0.84) | 7.17e-06 | 0.07 |
| Kidney Disease or Failure | 0.85 (0.72, 1.00) | 0.05 | 0.08 | 0.90 (0.76, 1.07) | 0.22 | 0.09 | 0.89 (0.75, 1.06) | 0.20 | 0.09 |
| Asthma | 0.70 (0.63, 0.78) | 2.58e-10 | 0.06 | 0.76 (0.67, 0.86) | 1.31e-05 | 0.06 | 0.77 (0.68, 0.87) | 4.36e-05 | 0.06 |
| Diabetes | 0.75 (0.69, 0.81) | 1.02e-12 | 0.04 | 0.82 (0.74, 0.91) | 1e-04 | 0.05 | 0.82 (0.74, 0.90) | 1e-04 | 0.05 |
| Cancer | 0.80 (0.74, 0.86) | 8.84e-09 | 0.04 | 0.89 (0.80, 0.98) | 0.02 | 0.05 | 0.90 (0.81, 0.99) | 0.04 | 0.05 |
| Other CMC | 0.78 (0.73, 0.84) | 9.60e-13 | 0.03 | 0.87 (0.78, 0.96) | 0.01 | 0.06 | 0.87 (0.78, 0.97) | 0.01 | 0.06 |
| Number of CMC |  |  |  |  |  |  |  |  |  |
| 0 |  |  |  | Ref | Ref | Ref | Ref | Ref | Ref |
| 1 |  |  |  | 0.96 (0.86, 1.08) | 0.54 | 0.06 | 0.96 (0.85, 1.08) | 0.48 | 0.06 |
| ≥2 |  |  |  | 0.86 (0.70, 1.05) | 0.14 | 0.10 | 0.85 (0.69, 1.04) | 0.11 | 0.10 |
| Care or Assistance Received by Type |  |  |  |  |  |  |  |  |  |
| Professional |  |  |  | 0.85 (0.76, 0.96) | 0.01 | 0.06 | 0.84 (0.75, 0.95) | 0.01 | 0.06 |
| Non-Professional |  |  |  | 1.01 (0.92, 1.11) | 0.86 | 0.05 | 1.00 (0.91, 1.10) | 0.95 | 0.05 |
| Healthcare Utilization by Type |  |  |  |  |  |  |  |  |  |
| Family Doctor Contact |  |  |  | 0.47 (0.41, 0.54) | < 2e-16 | 0.07 | 0.48 (0.42, 0.54) | < 2e-16 | 0.07 |
| Specialist Contact |  |  |  | 0.78 (0.73, 0.84) | 4.02e-12 | 0.04 | 0.79 (0.74, 0.85) | 6.47e-11 | 0.04 |
| Hospitalization History |  |  |  | 1.06 (0.95, 1.19) | 0.27 | 0.06 | 1.07 (0.95, 1.19) | 0.27 | 0.06 |
| Self-Rated Health |  |  |  |  |  |  |  |  |  |
| Excellent |  |  |  |  |  |  | Ref | Ref | Ref |
| Very Good |  |  |  |  |  |  | 0.91 (0.83, 1.00) | 0.04 | 0.05 |
| Good |  |  |  |  |  |  | 0.89 (0.80, 0.98) | 0.02 | 0.05 |
| Fair |  |  |  |  |  |  | 0.92 (0.80, 1.05) | 0.21 | 0.07 |
| Poor |  |  |  |  |  |  | 0.75 (0.58, 0.97) | 0.03 | 0.13 |
| Number in Household |  |  |  |  |  |  |  |  |  |
| 0 |  |  |  |  |  |  | Ref | Ref | Ref |
| 1 |  |  |  |  |  |  | 0.98 (0.90, 1.06) | 0.59 | 0.04 |
| ≥ 2 |  |  |  |  |  |  | 1.28 (1.13, 1.45) | 7.13e-05 | 0.06 |
| Exercise |  |  |  |  |  |  |  |  |  |
| None or Seldom |  |  |  |  |  |  | Ref | Ref | Ref |
| Sometimes or Often |  |  |  |  |  |  | 0.98 (0.90, 1.07) | 0.67 | 0.04 |
| Current Smoking |  |  |  |  |  |  |  |  |  |
| Not at All |  |  |  |  |  |  | Ref | Ref | Ref |
| Occasionally |  |  |  |  |  |  | 1.14 (0.81, 1.60) | 0.46 | 0.17 |
| Daily |  |  |  |  |  |  | 1.55 (1.33, 1.81) | 1.36e-08 | 0.08 |
| Alcohol |  |  |  |  |  |  |  |  |  |
| Never |  |  |  |  |  |  | Ref | Ref | Ref |
| Occasionally |  |  |  |  |  |  | 1.04 (0.92, 1.17) | 0.54 | 0.06 |
| Regular |  |  |  |  |  |  | 0.83 (0.76, 0.91) | 1e-04 | 0.05 |

*^a^Grey cells indicate variables that were not included in the model represented by that column.*

**Table 2. Regression Analysis Output: Canadian residents aged 46-64 years with at least one chronic medical condition (CMC) (N=** **11,250)**

|  | Model 1^a^  N=10,551 | | | Model 2^a^  N=10,522 | | | Model 3^a^  N=10,502 | | |
| --- | --- | --- | --- | --- | --- | --- | --- | --- | --- |
|  | aOR  (95% CI) | p-value | Standard Error | aOR  (95% CI) | p-value | Standard Error | aOR  (95% CI) | p-value | Standard Error |
| Age (years) |  |  |  |  |  |  |  |  |  |
| 55-64 | Ref | Ref | Ref | Ref | Ref | Ref | Ref | Ref | Ref |
| 46-54 | 1.44 (1.31, 1.58) | 2.74e-14 | 0.05 | 1.43 (1.30, 1.57) | 1.05e-13 | 0.05 | 1.37 (1.25, 1.51) | 1.52e-10 | 0.05 |
| Province of Residence |  |  |  |  |  |  |  |  |  |
| Ontario | Ref | Ref | Ref | Ref | Ref | Ref | Ref | Ref | Ref |
| Newfoundland | 1.34 (1.13, 1.58) | 8e-04 | 0.09 | 1.39 (1.17, 1.64) | 2e-04 | 0.09 | 1.39 (1.17, 1.65) | 2e-04 | 0.09 |
| Prince Edward Island | 0.83 (0.60, 1.14) | 0.24 | 0.16 | 0.80 (0.58, 1.11) | 0.19 | 0.16 | 0.80 (0.58, 1.11) | 0.18 | 0.17 |
| Nova Scotia | 0.56 (0.47, 0.66) | 2.86e-11 | 0.09 | 0.57 (0.48, 0.67) | 9.33e-11 | 0.09 | 0.57 (0.48, 0.68) | 1.70e-10 | 0.09 |
| New Brunswick | 1.03 (0.79, 1.33) | 0.85 | 0.13 | 1.05 (0.80, 1.36) | 0.74 | 0.14 | 1.09 (0.84, 1.43) | 0.52 | 0.14 |
| Quebec | 2.27 (2.00, 2.58) | < 2e-16 | 0.06 | 2.23 (1.96, 2.53) | < 2e-16 | 0.06 | 2.21 (1.95, 2.51) | < 2e-16 | 0.07 |
| Manitoba | 1.08 (0.93, 1.27) | 0.32 | 0.08 | 1.09 (0.93, 1.27) | 0.31 | 0.08 | 1.09 (0.93, 1.28) | 0.27 | 0.08 |
| Saskatchewan | 1.13 (0.86, 1.49) | 0.39 | 0.14 | 1.13 (0.85, 1.50) | 0.39 | 0.14 | 1.13 (0.85, 1.50) | 0.39 | 0.14 |
| Alberta | 0.84 (0.72, 0.97) | 0.02 | 0.07 | 0.84 (0.72, 0.97) | 0.02 | 0.08 | 0.84 (0.72, 0.97) | 0.02 | 0.08 |
| British Columbia | 1.09 (0.96, 1.23) | 0.17 | 0.06 | 1.10 (0.97, 1.25) | 0.13 | 0.06 | 1.12 (0.99, 1.26) | 0.09 | 0.06 |
| Sex |  |  |  |  |  |  |  |  |  |
| Female | Ref | Ref | Ref | Ref | Ref | Ref | Ref | Ref | Ref |
| Male | 1.22 (1.12, 1.32) | 1.87e-06 | 0.04 | 1.20 (1.10, 1.30) | 1.54e-05 | 0.04 | 1.18 (1.09, 1.28) | 8.77e-05 | 0.04 |
| Urbanicity |  |  |  |  |  |  |  |  |  |
| Urban | Ref | Ref | Ref | Ref | Ref | Ref | Ref | Ref | Ref |
| Rural | 1.30 (1.16, 1.47) | 9.80e-06 | 0.06 | 1.28 (1.13, 1.44) | 5.25e-05 | 0.06 | 1.30 (1.15, 1.46) | 2.15e-05 | 0.06 |
| Household Income (Canadian dollars) |  |  |  |  |  |  |  |  |  |
| < 20000 | Ref | Ref | Ref | Ref | Ref | Ref | Ref | Ref | Ref |
| ≥20000-<50000 | 1.02 (0.83, 1.26) | 0.82 | 0.11 | 1.00 (0.81, 1.24) | 0.99 | 0.11 | 1.00 (0.81, 1.25) | 0.98 | 0.11 |
| ≥50000-<100000 | 0.90 (0.73, 1.09) | 0.28 | 0.1 | 0.87 (0.71, 1.07) | 0.19 | 0.10 | 0.88 (0.71, 1.08) | 0.22 | 0.11 |
| ≥100000-<150000 | 0.92 (0.75, 1.13) | 0.44 | 0.11 | 0.90 (0.73, 1.11) | 0.33 | 0.11 | 0.90 (0.71, 1.12) | 0.34 | 0.12 |
| ≥150000 | 0.71 (0.58, 0.88) | 2e-03 | 0.11 | 0.70 (0.56, 0.86) | 9e-04 | 0.11 | 0.69 (0.55, 0.87) | 2e-03 | 0.12 |
| Education |  |  |  |  |  |  |  |  |  |
| Less than secondary school graduation | Ref | Ref | Ref | Ref | Ref | Ref | Ref | Ref | Ref |
| Secondary school graduation, no post-secondary education | 0.89 (0.69, 1.14) | 0.36 | 0.13 | 0.89 (0.69, 1.14) | 0.36 | 0.13 | 0.88 (0.68, 1.13) | 0.32 | 0.13 |
| Some post-secondary education | 0.87 (0.67, 1.13) | 0.31 | 0.13 | 0.88 (0.68, 1.15) | 0.36 | 0.13 | 0.87 (0.67, 1.13) | 0.30 | 0.14 |
| Post-secondary degree/diploma | 0.77 (0.62, 0.96) | 0.02 | 0.11 | 0.88 (0.62, 0.98) | 0.03 | 0.11 | 0.78 (0.62, 0.98) | 0.03 | 0.12 |
| Race |  |  |  |  |  |  |  |  |  |
| White | Ref | Ref | Ref | Ref | Ref | Ref | Ref | Ref | Ref |
| Non-White | 1.16 (0.98, 1.38) | 0.08 | 0.09 | 1.16 (0.98, 1.38) | 0.09 | 0.09 | 1.15 (0.96, 1.36) | 0.12 | 0.09 |
| CMC by Type |  |  |  |  |  |  |  |  |  |
| Heart Disease | 0.77 (0.68, 0.89) | 2e-04 | 0.07 | 0.90 (0.77, 1.04) | 0.16 | 0.08 | 0.91 (0.78, 1.06) | 0.23 | 0.08 |
| Lung Problems | 0.76 (0.66, 0.88) | 2e-04 | 0.07 | 0.87 (0.74, 1.02) | 0.09 | 0.08 | 0.84 (0.72, 0.99) | 0.04 | 0.08 |
| Kidney Disease or Failure | 0.79 (0.65, 0.98) | 0.03 | 0.11 | 0.91 (0.73, 1.12) | 0.37 | 0.11 | 0.92 (0.74, 1.14) | 0.45 | 0.110004 |
| Asthma | 0.69 (0.63, 0.76) | 1.25e-13 | 0.05 | 0.80 (0.70, 0.90) | 5e-04 | 0.06 | 0.81 (0.71, 0.92) | 1.4e-03 | 0.07 |
| Diabetes | 0.79 (0.72, 0.87) | 3.14e-07 | 0.05 | 0.91 (0.80, 1.03) | 0.15 | 0.06 | 0.93 (0.82, 1.05) | 0.25 | 0.07 |
| Cancer | 0.82 (0.73, 0.91) | 2e-04 | 0.05 | 0.98 (0.85, 1.12) | 0.75 | 0.07 | 0.98 (0.86, 1.13) | 0.80 | 0.07 |
| Other CMC | 0.74 (0.67, 0.80) | 1.11e-11 | 0.05 | 0.86 (0.76, 0.98) | 0.02 | 0.06 | 0.87 (0.77, 0.99) | 0.04 | 0.07 |
| Number of CMC |  |  |  |  |  |  |  |  |  |
| ≥ 2 |  |  |  | Ref | Ref | Ref | Ref | Ref | Ref |
| 1 |  |  |  | 1.21 (1.04, 1.42) | 0.01 | 0.08 | 1.23 (1.05, 1.43) | 0.01 | 0.08 |
| Care or Assistance Received by Type |  |  |  |  |  |  |  |  |  |
| Professional |  |  |  | 0.79 (0.64, 0.96) | 0.02 | 0.10 | 0.81 (0.66, 0.99) | 0.04 | 0.10 |
| Non-Professional |  |  |  | 0.92 (0.81, 1.04) | 0.18 | 0.06 | 0.93 (0.82, 1.06) | 0.27 | 0.06 |
| Healthcare Utilization by Type |  |  |  |  |  |  |  |  |  |
| Family Doctor Contact |  |  |  | 0.53 (0.45, 0.62) | 5.34e-15 | 0.08 | 0.53 (0.45, 0.62) | 1.15e-14 | 0.08 |
| Specialist Contact |  |  |  | 0.77 (0.70, 0.84) | 2.11e-09 | 0.04 | 0.78 (0.72, 0.85) | 3.38e-08 | 0.04 |
| Hospitalization History |  |  |  | 1.11 (0.95, 1.30) | 0.19 | 0.08 | 1.12 (0.96, 1.31) | 0.16 | 0.08 |
| Self-Rated Health |  |  |  |  |  |  |  |  |  |
| Excellent |  |  |  |  |  |  | Ref | Ref | Ref |
| Very Good |  |  |  |  |  |  | 1.06 (0.93, 1.20) | 0.41 | 0.07 |
| Good |  |  |  |  |  |  | 1.03 (0.89, 1.18) | 0.71 | 0.07 |
| Fair |  |  |  |  |  |  | 0.92 (0.77, 1.10) | 0.37 | 0.09 |
| Poor |  |  |  |  |  |  | 0.80 (0.60, 1.07) | 0.14 | 0.15 |
| Number in Household |  |  |  |  |  |  |  |  |  |
| 0 |  |  |  |  |  |  | Ref | Ref | Ref |
| 1 |  |  |  |  |  |  | 0.95 (0.84, 1.08) | 0.43 | 0.06 |
| ≥ 2 |  |  |  |  |  |  | 1.13 (0.98, 1.29) | 0.08 | 0.07 |
| Exercise |  |  |  |  |  |  |  |  |  |
| None or Seldom |  |  |  |  |  |  | Ref | Ref | Ref |
| Sometimes or Often |  |  |  |  |  |  | 0.95 (0.86, 1.04) | 0.27 | 0.05 |
| Current Smoking |  |  |  |  |  |  |  |  |  |
| Not at All |  |  |  |  |  |  | Ref | Ref | Ref |
| Occasionally |  |  |  |  |  |  | 1.22 (0.92, 1.63) | 0.17 | 0.15 |
| Daily |  |  |  |  |  |  | 1.56 (1.33, 1.83) | 3.38e-08 | 0.08 |
| Alcohol |  |  |  |  |  |  |  |  |  |
| Never |  |  |  |  |  |  | Ref | Ref | Ref |
| Occasionally |  |  |  |  |  |  | 0.94 (0.80, 1.11) | 0.48 | 0.08 |
| Regular |  |  |  |  |  |  | 1.07 (0.93, 1.22) | 0.34 | 0.07 |

*^a^Grey cells indicate variables that were not included in the model represented by that column.*
